# Multiple region whole-exome sequencing reveals dramatically evolving intratumor genomic heterogeneity in esophageal squamous cell carcinoma

**DOI:** 10.1038/oncsis.2015.34

**Published:** 2015-11-30

**Authors:** W Cao, W Wu, M Yan, F Tian, C Ma, Q Zhang, X Li, P Han, Z Liu, J Gu, F G Biddle

**Affiliations:** 1 Translational Medical Center, Zhengzhou Central Hospital Affiliated to Zhengzhou University, Zhengzhou, China; 2 Department of Pathology and Laboratory Medicine, Arnie Charbonneau Cancer Institute, Cumming School of Medicine, University of Calgary, Calgary Alberta, Canada; 3 Medical School, Zhengzhou University, Zhengzhou, China; 4 Linzhou Cancer Hospital, Linzhou, China; 5 Science and Education Department, Health Bureau of Zhengzhou, Zhengzhou, China; 6 Departments of Medical Genetics and Biological Sciences, University of Calgary, Calgary, Canada

## Abstract

Cancer is a disease of genome instability and genomic alterations; now, genomic heterogeneity is rapidly emerging as a defining feature of cancer, both within and between tumors. Motivation for our pilot study of tumor heterogeneity in esophageal squamous cell carcinoma (ESCC) is that it is not well studied, but the highest incidences of esophageal cancers are found in China and ESCC is the most common type. We profiled the mutations and changes in copy number that were identified by whole-exome sequencing and array-based comparative genomic hybridization in multiple regions within an ESCC from two patients. The average mutational heterogeneity rate was 90% in all regions of the individual tumors in each patient; most somatic point mutations were nonsynonymous substitutions, small Indels occurred in untranslated regions of genes, and copy number alterations varied among multiple regions of a tumor. Independent Sanger sequencing technology confirmed selected gene mutations with more than 88% concordance. Phylogenetic analysis of the somatic mutation frequency demonstrated that multiple, genomically heterogeneous divergent clones evolve and co-exist within a primary ESCC and metastatic subclones result from the dispersal and adaptation of an initially non-metastatic parental clone. Therefore, a single-region sampling will not reflect the evolving architecture of a genomically heterogeneous landscape of mutations in ESCC tumors and the divergent complexity of this genomic heterogeneity among patients will complicate any promise of a simple genetic or epigenetic diagnostic signature in ESCC. We conclude that any potential for informative biomarker discovery in ESCC and targeted personalized therapies will require a deeper understanding of the functional biology of the ontogeny and phylogeny of the tumor heterogeneity.

## Introduction

Cancer is a disease of genome instability and a resulting accumulation of genetic and epigenetic alteration. Global cancer genome projects have contributed to the identification and molecular classification of hundreds of cancer genes and the genomic architecture. With a deepening of the sequencing of the cancer genome, genomic heterogeneity is rapidly emerging as a defining feature of cancer, not only between tumors, but also within tumors. Accumulating evidence for intratumor heterogeneity^1, 2, 3, 4, 5, 6^ is showing that tumors evolve through a process of branched evolution with genetically distinct subclones, which lead to tumor recurrence, drug resistance and metastatic potential.^7, 8^

Esophageal squamous cell carcinoma (ESCC) is the most common histological subtype of esophageal cancer in South-Eastern and Central Asia, particularly in China.^9^ Large-scale genome sequencing of ESCC has identified known frequently mutated genes, such as TP53, and other previously unrecognized mutated genes,^10, 11, 12^ but intratumor heterogeneity in ESCC has not been well studied. Therefore, to establish the framework for a comprehensive and systematic whole-genome analysis of ESCC, we conducted a pilot study in ESCC patients, using multiple-region, whole-exome sequencing and array-based comparative genomic hybridization (aCGH), applied to 11 tumor regions from two surgically resected ESCCs, including metastatic lymph nodes. We show here that each tumor region has substantial genomic heterogeneity with its own unique profile of mutations and copy number alterations. Each tumor region was characterized for non-silent mutations and a branching evolution of cancer development was inferred. Functional analysis revealed common as well as unique, actionable and druggable mutated genes in the landscape of intratumor genomic heterogeneity in ESCC. Therefore, we will suggest that targeting the gene regulatory networks, which underlie the fitness landscape of ESCC, with a combinatorial approach rather than current canonical protocols may be necessary for optimal tumor treatment and control.

## Results

### Mutational profile in multiple regions of ESCC

**Figure 1:**
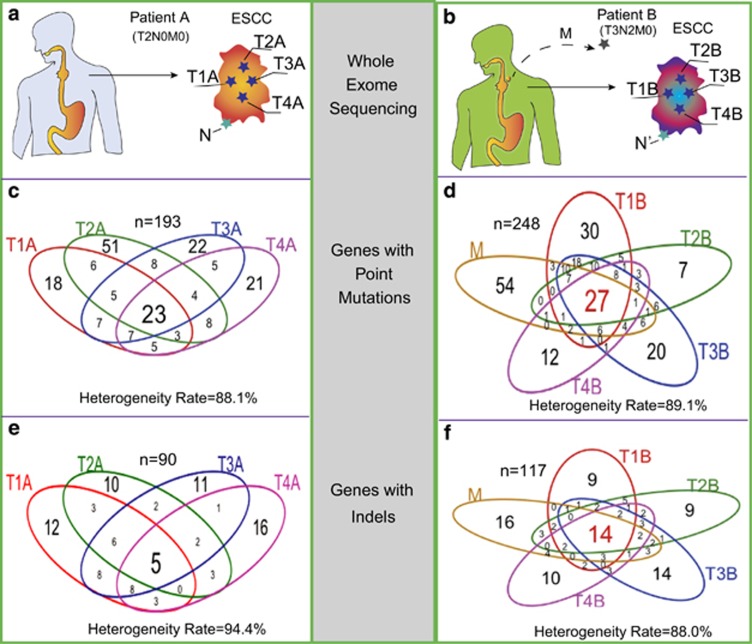
Intratumor heterogeneity of somatic mutations in ESCC. (**a**, **b**) Four spatially separated samples were obtained from a surgically resected ESCC as well as non-tumor tissue and associated metastatic lymph nodes were collected for multi-region exome sequencing and aCGH assay. (**c**, **d**) The Venn diagrams show the number of genes in each tumor region with SPMs from PtA and PtB. (**e**, **f**) The number of genes affected with insertions and deletions (Indels) in each region from PtA and PtB. The heterogeneity rate was computed with the total affected genes divided by the number of affected genes not shared by all tumor regions.

To characterize the extent of intratumor genomic heterogeneity in ESCC, we performed whole-exome sequencing on four separate regions within a primary ESCC sample and a non-tumor region from adjacent normal tissue from two patients (Pt), denoted as PtA and PtB (Figures 1a and b, Supplementary Table S1). PtA was diagnosed with ESCC without lymph node invasion, clinical stage was T2N0M0; PtB was diagnosed with advanced ESCC with lymph node metastasis, clinical stage was T3N2M0. Genomic aberrations in these samples were assessed for somatic point mutations (SPMs) and somatic small insertions or deletions (Indels). Annotation of the SPMs were categorized as nonsynonymous or missense mutations, synonymous or silent mutations, stop-gain or nonsense mutations, and splice site mutations. The non-silent mutations (nonsynonymous, stop-gain and splice site mutations) comprise more than 50% of the defined mutations in each tumor region (Supplementary Figures S1c and d). Indels occurred in exons of genes resulting in frameshift or non-frameshift mutations, as well as in the untranslated regions (5' or 3'-UTRs) and splice sites of genes. The majority of Indels were in UTRs (Supplementary Figures S1e and f). The predominant type of mutation in both patients is a C>T/G>A transition; the second most frequent mutation is an A>G/T>C transition in PtA, and a C>G/G>C transversion in PtB (Supplementary Figure S2). The difference in the mutation spectra between the two patients may be due to a change in or a reflection of different mutational mechanisms during ESCC cancer development.^12, 13^

We then asked what genes are affected by the identified mutations across the tumor regions in each patient. Overall, only 23 of 193 genes with SPMs and 5 of 90 genes with Indels in PtA (Figures 1c and e) were found in all four regions, accounting for 12% and 7% common mutated genes with SPMs and Indels, respectively. Similarly, all tumor regions in PtB, including the metastasis, shared only 11% of the 248 mutated genes with SPMs and 12% of the 117 mutated genes with Indels (Figures 1d and f). Therefore, one measure of the degree of intratumor heterogeneity is the heterogeneity rate and it is defined as the number of mutated genes that are detected in one or more, but not all regions of a tumor. Both patients have similar average intratumor heterogeneity rates of approximately 90% for mutated genes with either SPMs or Indels.

### Copy number alterations in multiple regions of ESCC

**Figure 2:**
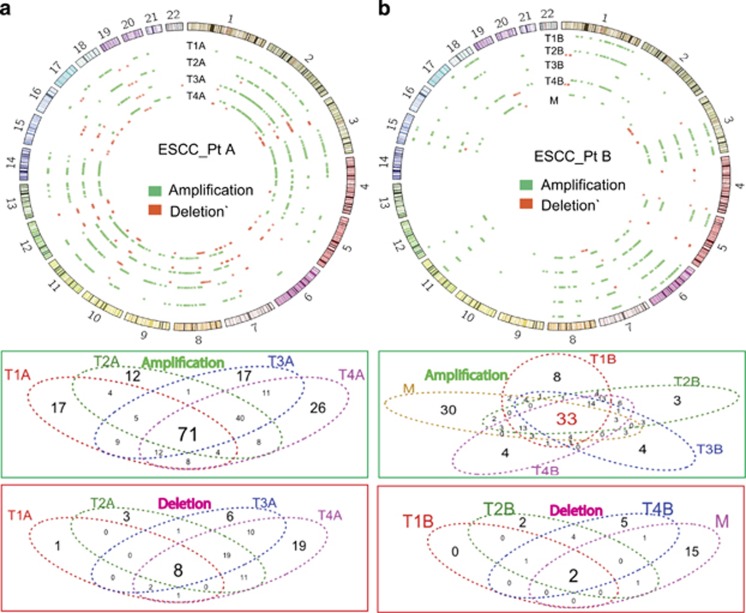
Intratumoral genomic copy number alterations of ESCC detected by aCGH. Genomic copy number alterations relative to non-tumor tissues in PtA (**a**) and in PtB (**b**) were plotted with Circos plot. Green color represents copy number amplification (Amp); Red color represents copy number deletion (Del), in each region of a given ESCC tissue. The total copy number alterations (Amp+Del): 403 in PtA, 262 in PtB. The Venn diagrams show the overlap of Amp or Del between each tumor region from PtA and PtB.

Copy number alterations in multiple regions of ESCC were surveyed using aCGH, and the amplifications and deletions of chromosomes were identified in each tumor region from PtA and PtB (Figure 2 and Supplementary Figures S3a and b). In PtA, 430 chromosomal segments were amplified or deleted in at least one region and among them, 71 (17%) genomic loci were amplified and 8 (2%) genomic loci were deleted in all four regions of the tumor (Figure 2a). The common amplified regions occurred in 2q, 3q, 8q, 10q, 11q, 14q and 18q, and the common deletions occurred in 3p21, 7q11.23, 10q24, 12q24, 19p13 and 19q13. All tumor regions did not share the majority of the chromosomal aberrations, indicating substantial intratumor chromosomal heterogeneity. In contrast, in PtB, only 262 chromosomal regions were amplified or deleted, in at least one region of the tumor (Figure 2b). There were 33 (13%) common amplified regions, located in 3p12, 3p11-13, 3q21.3-3q26.31, 6q, 7q, 8q and Xq, and two common deletions, which occurred at 4q35.2 and Yp11.31-Yq12, were shared only in tumor regions T1B, T2B, T4B and M (no deletion was detected in tumor region T3B). We note that the known frequent somatic CNVs, involving 3q26 (PRKCI gene amplification),^14^ 9p21 (SOX2 gene amplification)^15^ and 11q13.3 (CCTN gene amplification),^16^ were detected in this study. When taken together, heterogeneous amplifications and deletions were observed in multiple regions of each ESCC with an average heterogeneity rate of approximately 92%, which is very similar to the heterogeneity rate observed for SPMs and Indels.

### Characterization of non-silent somatic mutations in multiple regions of ESCC

**Figure 3:**
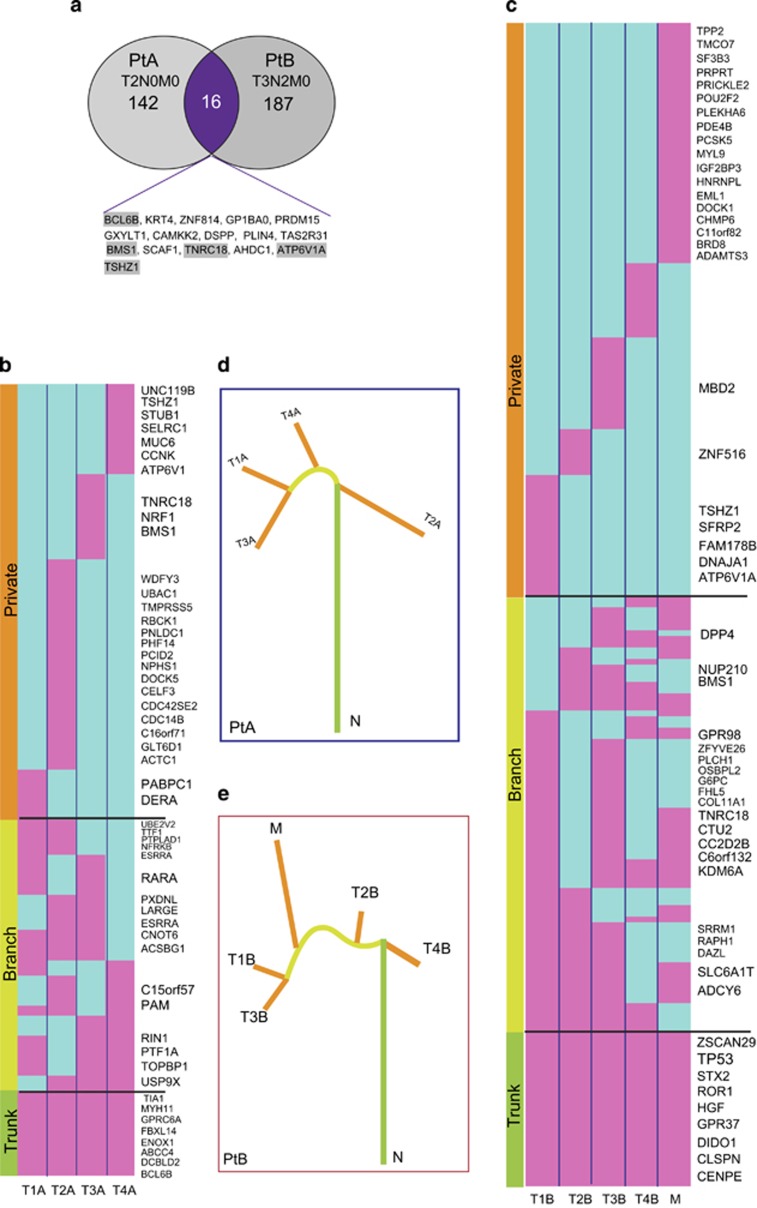
Characterization of non-silent mutations in multiple regional samples of ESCC. (**a**) Overlap of non-silent mutation affected genes between PtA (*n*=158) and PtB (*n*=203). (**b**, **c**) Heatmaps show the regional distributions of non-silent mutations in PtA and PtB; presence (pink), absence (light blue) is indicated for each tumor region. Gene mutations in all regions of a tumor shown with green bar, designated as ‘Trunk' gene mutations in some regions of a tumor shown with yellow bar (‘Branch'); gene mutations in only one regions of a tumor shown with orange bar (‘Private'), mutated cancer genes are listed on the right of the heatmaps. (**d**, **e**) Phylogenetic trees generated using the Ape and Phangorn packages in R based on the distribution of all detected mutations; trunk and branch lengths are proportional to the number of non-silent mutations in each tumor region.

The nonsynonymous mutations, stop-gain mutations, splice site mutations and other mutations, including small Indels (frameshift and non-frameshift), generally change the protein function and we grouped them collectively as non-silent mutations. We note that the number of genes affected by mutations is relatively less than the total number of mutations, owing to the fact that multiple mutations may occur in the same gene. On the basis of this classification, there were 158 and 203 non-silent mutated genes (present in at least one tumor region) in PtA and PtB, respectively (Supplementary Tables S2 and S3), but only 16 genes were common to both patients (Figure 3a). We selected 51 gene mutations, including SPM and Indels, for independent validation by a Sanger sequencing approach, and 46 out 51 gene mutations (88%) were confirmed (Supplementary Table S4).

To characterize intratumor heterogeneity of the non-silent mutated genes, we used the ‘trunk' to represent ubiquitous mutations present in all regions of a tumor, the ‘branch' to stand for heterogeneous mutations present in some, but not all regions of the tumor, and ‘private' to correspond to mutations that are present in only one region of a tumor. We mapped these non-silent mutated genes across all spatially separated regions shown in the heatmap in Figures 3b and c. In PtA, we classified 158 non-silent mutated genes into 17 trunk gene mutations, 54 branch gene mutations and 87 private mutations. In PtB, 27 of 203 mutant genes were in the trunk, 76 mutant genes were located in the branch section and 100 genes were private mutations. Detection of private mutations in all tumor regions suggests that an ongoing, regional clonal evolution is occurring in PtA and PtB.

To determine whether these non-silent mutated genes are associated with cancer, we searched for our identified mutant genes in the COSMIC (Catalog Of Somatic Mutations In Cancer) database and other cancer-related studies.^10, 11, 12^ There are 52 recognized cancer gene mutations across the tumor regions in PtA and 55 recognized cancer gene mutations across the tumor regions in PtB, and they are listed on the right side of the respective heatmaps in Figures 3b and c. In the trunk of the heatmap, 8 of the 17 mutated genes in PtA and 9 of the 27 mutated genes in PtB are known cancer-associated genes. PtB is a more advanced case with adjacent lymph node metastasis and we note that the TP53 (C85X, C85Y) mutation was detected in all tumor regions of PtB (but not PtA) and the metastatic region (M) of PtB has 18 cancer-associated gene mutations.

Tumor evolution in each patient was reconstructed from the regional mutation frequencies using a phylogenetic algorithm.^17^ A branching rather than a linear tumor evolutionary pattern was inferred for each patient and is illustrated in Figures 3d and e. Subclonal populations, with variable evolutionary distances between them, co-exist within a tumor and share trunk mutations. As well, the subclonal populations harbor their own private mutations, while continuing to maintain a stable phenotype or cancer property.

### Characterization of mutations in UTRs from multiple regions of ESCC

We assessed SPMs and Indels in UTRs (3' and 5') that may change the regulatory elements of genes. UTRs contain binding sites for microRNAs and mutations in those UTRs may perturb interactions between microRNAs and their target genes and, as a consequence, disrupt regulatory functions of the microRNAs. PtA had a total of 232 detected mutations in UTRs and PtB had a total of 366. We retrieved 1858 microRNA-binding sites that were predicted with the miRase algorithm and used them as a database to computationally assess the identified mutations in UTRs (Supplementary Figure S4). Two genes, CTDSPL2 (Chr15:44818173-44818173, an ‘A' insertion) in region T3A and BCL7A (Chr12: 122498799-122498799, an ‘A' insertion) in region T4A, were predicted to have mutations in 3'-UTR disrupting microRNA-binding sites that are recognized by miR129/129-5p and miR204/211, respectively. The UTR mutations in PtB were not predicted to affect known microRNA-binding sites. Nevertheless, the majority of identified UTR mutations in both PtA and PtB appear to be of unknown significance.

### Divergent complexity of functional intratumor genomic heterogeneity

**Figure 4:**
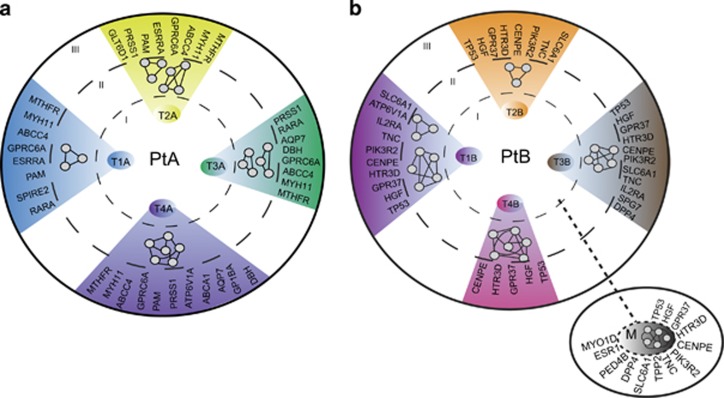
Functional intratumor genomic heterogeneity in ESCC. Non-silent mutations in each tumor region (circle I) were subjected to KEGG and Interactom database search using GlueGo algorithm. The networks of pathways and biological processes (summarized in Table 1) in each tumor region are illustrated conceptually as nodes and edges (circle II). The actionable and druggable genes in each tumor region were predicted with the Drug-Gene Interaction DATABASE (http://dgidb.genome.wustl.edu/) (circle III). Left panel: PtA, right panel: PtB; M, Metastasis.

Evolving intratumor mutational heterogeneity in ESCC reveals a complex divergence of misregulation of cancer-cell signaling pathways and the intratumor mutational heterogeneity may provide an informed guidance of combinatorial therapy. Most mutated genes do express and produce abnormal proteins and they may be components of different cancer pathways. Therefore, we made two functional assessments of the multiple tumor regions. First, we made functional predictions with GO terms in the KEGG and REACTOM search algorithms applied to the profiles of the non-silent mutations in the different tumor regions (Supplementary Figure S4). Functional heterogeneity in the different tumor regions suggested different regulatory signaling and cellular metabolic pathways (Table 1). The inferred gene regulatory networks of each tumor region from PtA and PtB, including the metastasis, are illustrated only symbolically with nodes and edges (circle II in Figures 4a and b). For example, MUC17, MUC5B and MUC6 gene mutations in tumor region T4A of PtA (Table 1) predict the perturbation of O-glycan biosynthesis and processing, and the GO terms in T4A differ from GO terms in other tumor regions. Similarly, mutations in the tumor regions from PtB suggest heterogeneous signaling pathways and biological processes, such as the HGF, PIK3R2 and TP53 mutations, which are known to result in cancer pathway activation. Mutations of genes CSN2, ESR1 and PIK3R2 may damage the prolactin-signaling pathway (Table 1). These data indicate that the divergent complexity of mutations, which is identified by genotyping of the separate tumor regions, may inevitably change the biological functionalities that are linked to intratumor heterogeneity.

Second, we used the well-established Drug-Gene Interaction Database (DGIDB) to predict actionable and druggable gene mutations. Drugs in this database are used clinically or they are currently in clinical trials. The results show that the patterns of potentially druggable mutated genes are heterogeneous in the different tumor regions. In PtA (circle III in Figure 4a), all tumor regions shared four common actionable mutated genes (MTFHR, MYH11, ABCC4 and GPRC6A); in PtB (circle III in Figure 4b), all tumor regions, including the metastasis, shared five druggable mutated genes (TP53, GPR37, HGF, CENPE and HTR3D). Perhaps more importantly, the tumor regions from each patient show an evolving divergent complexity of additional actionable and druggable mutated genes.

## Discussion

Single biopsy sampling for molecular analysis of tumors from patient cohorts is a standard analytical method and it is effective for diagnosis of single gene disorders. However, single tumor biopsy samples do not reveal the complexity of the genomic landscape in tumors with intratumor heterogeneity.^1, 2, 3, 5, 18^ Therefore, multiple regional sampling of tumors to estimate intratumor genomic heterogeneity has emerged as a non-canonical protocol to characterize actionable targets, make treatment decisions and manage chemo-resistance.^7^

In our pilot study, we assessed multiple regional samples of two primary ESCC tumors and an associated metastasis with whole-exome sequencing and aCGH. We found extensive divergent mutational profiles of SPMs, Indels, copy number amplifications and deletions in the spatially separated samples within each tumor and metastasis with an average intratumor heterogeneity rate 90% (Figures 1 and 2). Further characterization of non-silent mutations in each tumor region showed only 17 (11%) out of 158 mutated genes in PtA (Figure 3b), and 27 (13%) of 203 in PtB (Figure 3c) to be common in all tumor regions (‘trunk' mutations). The overall percentage of shared mutations between multiple tumor regions of ESCC in this study is dramatically lower than in other types of cancer such as clear cell renal cell carcinoma (30~35%)^1, 2^ and high-grade serous ovarian cancers (52%).^4^ In other words, the rate of intratumor genomic heterogeneity is higher in ESCC (~90%) than in other types of tumor, including esophageal adenocarcinoma (56%).^6^ Higher intratumor heterogeneity index in esophageal adenocarcinoma is strongly correlated with poor responses to neoadjuvant chemotherapy.^6^ We suggest that our observed high intratumor heterogeneity rate in ESCC may contribute to its poor overall 5-year survival rates of 15–25%.^19^

To understand the biological significance of the non-silent mutation-affected genes that we detected in ESCC, we compared our mutations from PtA and PtB with the COSMIC database and other ESCC genome sequencing studies. Approximately one-third of our non-silent mutated genes are cancer-related. For example, the mutated genes common in all tumor regions from PtA, BCL6B,^20^ MYH11,^21^ SUFU^22^ act as tumor suppressor genes, and mutations in BCL6B (Indel), MYH11(M816K) and SUFU(Y90S) could inactivate the gene function and contribute to tumorigenesis. Over expression of DCBLD2^23^ may act as an oncogene interacting with the EGFR and PI3K/Akt signaling pathways; gain of function of DCBLD2 mutation (Indel) could participate with cellular proliferative pathways to promote tumor progression. In contrast, PtB is a more advanced case of ESCC with an adjacent lymph node metastasis and, as might be expected, more mutated genes were identified in PtB, but the intratumor genomic heterogeneity rate was the same as in PtA. We note that TP53 is a frequently and early mutated cancer gene,^6^ but it was detected as a trunk mutation only in PtB. CENPE is another trunk mutation gene in PtB and normally, it has a key role in the movement of chromosomes toward the metaphase plate during mitosis; the observed CENPE (Q1302E) gene mutation may disrupt its normal function and lead to chromosomal instability, but again, it only was found in PtB.

UTRs of genes contain microRNA regulatory-binding sites and mutations in these sequences could alter miRNA::mRNA interaction and significantly alter gene expression. These mutations in UTRs and the dysregulation of their functions are usually not reported in current studies of intratumor heterogeneity. In our computational search, we found multiple microRNA-binding sites mutated in target genes CTDSPL2 in region T3A and BCL7A in region T4A from PtA. These microRNAs are miR129-5p,^24, 25^ miR204^26^ and miR211,^27^ which are putative tumor-suppressor microRNAs. These types of mutation in gene regulatory elements provide a hypothesis and a foundation for further experimental investigations.

Phylogenetic analysis is clearly a useful approach to visualize the complexity of intratumor heterogeneity as a tree structure in a given tumor because it summarizes the ontogeny of genomic similarity and divergence in all tumor regions. The present analysis unequivocally indicates that clonal subpopulations co-exist in the primary tumor and a metastatic subpopulation may be derived by additional mutations from initially non-metastatic parental clones in the primary tumor. This observation is supported by similar studies of intratumor heterogeneity in pancreatic cancer^18^ and clear cell renal cell cancer.^1^

An intratumor heterogeneous genomic architecture may generate a microenvironment with divergent heterogeneous cellular signaling pathways and biological processes, which may harbor various actionable and druggable mutated genes. Therefore, we assessed the non-silent genes in each tumor region for potential regional specific functional pathways (Table 1) and explored the potential for regional targeted therapy with small molecules (Figure 4). In PtA, no common pathways were identified, but there are four common actionable mutated genes, which are MHFR, MYH11, ABCC4 and GRPC6A. In PtB, a cancer pathway (melanoma) was identified in all tumor regions except T4B, and this pathway is composed of TP53, HGF and PIK3R2, which have existing targeted drugs. However, each tumor region also has its own uniqueness of cellular pathways and available targeted mutated genes. Thus, the functional intratumor heterogeneity presents a challenge to precision medicine. To achieve optimal tumor treatment and tumor control, it may be necessary to target clonally dominant truncal somatic events or adopt multiple targeted therapies in a combinatorial approach.^28^

In summary, our observations, along with other studies, demonstrate that intratumor heterogeneity is a hallmark of ESCC, as it is for most cancers. Therefore, multi-regional sampling is a more informative approach for diagnosis, for potential biomarker discovery and for making treatment decisions. In addition, the evolving, divergent complexity of intratumor genomic and functional heterogeneity points to a necessity for unconventional strategies to understand the logic of the ESCC genomic system for reprogramming of its gene-regulatory network.

## Materials and methods

### Specimens

Written informed consent was obtained from two male patients before surgery and the Institutional Review Board for the use of human subjects at Zhengzhou Central Hospital, affiliated to Zhengzhou University, approved our study protocol. Multiregional specimens from primary tumors were obtained from two patients with ESCC who underwent surgical treatment and adjacent non-neoplastic tissues were obtained as references. One metastatic specimen was also obtained from one of the patients. Basic patient information is listed in Supplementary Table S1. Specimens were frozen in liquid nitrogen immediately after surgical resection. Neither patient had prior chemotherapy or radiotherapy, nor did they have any other serious disease. All ESCC tissues were histopathologically diagnosed by at least two independent senior pathologists.

### Whole-exome sequencing

The genomic DNA from tumors of multiple regions, metastatic areas and matched adjacent normal tissues from the two ESCC patients were sheared by Bioruptor_NGS (Diagenode SA, Denville, NJ, USA) to produce fragment sizes of 200~300 bp. Samples of 100 ng of purified DNA were carried out through a process of end repair, phosphorylation and ligation to barcoded sequencing adapters. Ligated DNA was size-selected for lengths between 200 and 350 bp and subjected to exonic hybrid capture using SeqCap EZ Human Exome Library v3.0 (Roche Nimblegen, Madison, WI, USA). Each of the captured libraries were multiplexed and sequenced on multiple Illumina HiSeq 2000 (Illumina, San Diego, CA, USA) flow cells to average target exome coverage of 50 × in neoplastic DNA and 60 × in reference tissue using 76- bp paired-end reads.

### Processing of sequencing data and detection of variation

After removing reads containing sequencing adapters and low-quality reads, high-quality reads were aligned to the NCBI reference genome (hg19) using Burrows-Wheeler Aligner 0.7.2 with default parameters. The SAMtools set of utilities was used to merge and remove duplicates. Local realignment and recalibration of base quality score was carried out with the GATK software package. SPMs (single nucleotide polymorphisms) and INDELs were predicted with GATK and variation file (*.vcf) was filtered and annotated with the ANNOVAR software tool. The sequence data have been deposited at the European Genome-Phenome Archive (EGA, http://www.ebi.ac.uk/ega/), which is hosted by the EMBL-EBI, under accession number EGAS 00001000965.

### aCGH processing and analysis of copy number

Each genomic DNA (0.5 μg) was fluorescently labeled with the NimbleGen enzymatic labeling protocol which uses Cy3 and Cy5-labeled random nanomers (TriLink Biotechnologies, San Diego, CA, USA), a heat fragmentation step at 98 °C for 10 min, and amplification with Klenow fragment 5'-3'exo- (NEB, Ipswich, MA, USA). Five micrograms of each Cy5-labeled sample were co-hybridized with 5 μg of gender matched Cy3-labeled human male or female reference DNA (Promega, Madison, WI, USA) on Agilent SurePrint G3 Human Catalog CGH 8 × 60K (Design ID 021924, Agilent, Santa Clara, CA, USA) following the hybridization and washing conditions from the Agilent Oligonucleotide Array-based CGH for Genomic DNA Analysis Protocol v6.2. Arrays were scanned with the Agilent DNA Microarray Scanner at a 3-μm scan resolution, and quantified with Feature Extraction 11.0.1.1. CGH-processed signal was then uploaded into Partek Suite software (v6.6) where data were visualized and analyzed. The amplifications and deletions were called with default parameters except for changing of minimum genomic markers=30 and signal/noise=0.5 to increase the stringency. The aCGH data have been deposited in National Center for Biotechnology Information (NCBI) Gene Expression Omnibus (GEO) and are accessible through (GEO) Series accession number GSE60625 (http://www.ncbi.nlm.nih.gov/geo/query/acc.cgi?acc=GSE60625).

### Phylogenetic analysis

Phylogenetic analysis was conducted with published methods^17^ in R (V 2.15.0) using the Ape and Phangorn packages. Briefly, genetic distances were estimated among different regions under a generalized Kimura model. The initial phylogenetic trees were created using a neighbor-joining algorithm. Likelihood optimization was then used to obtain the maximum likelihood tree for each region. Finally, a non-parametric bootstrap on each maximum likelihood tree was conducted to estimate the support for individual branches (*n*=1000 iterations)

### Functional analysis of non-silent mutations

The mutations (SPMs and Indels) in each tumor region obtained from whole-exome sequencing were further analyzed with the R project for statistical computing (version 3.1.2) and customized script (Supplementary Figure S3). The Cytoscape (v3.1.1) with GlueGo plugin was used for pathways analysis. The DGIDB (http://dgidb.genome.wustl.edu/) was used to predict actionable and druggable mutated genes.

## Figures and Tables

**Table 1:** Pathways in each tumor region of ESCC

	*GO terms*	P *value*	*Associated genes*
*PtA*			
T1A	Intracellular estrogen receptor signaling pathway	3.00E-04	CRIPAK, POU4F2, RARA
T2A	Secretory granule membrane	2.30E-03	ABCC4, PAM, PCSK4
	DNA packaging complex	1.60E-03	H1FOO, HIST1H3D, PXDNL, SMC4
T3A	Myosin complex	2.50E-03	MYH11, MYO18A, MYO18B
	Negative regulation of translation	1.80E-03	EIF2AK4, RARA, TIA1
T4A	O-linked glycosylation of mucins	9.80E-04	
	Termination of O-glycan biosynthesis	3.00E-04	MUC17, MUC5B, MUC6
	O-glycan processing	2.20E-03	
	Sectetory granule membrane	1.60E-03	ABCC4, DBH, PAM

*PtB*			
T1B	Transport of mature transcript to cytoplasm	9.10E-03	NUP153, NXF1, SRRM1
	Transport of mature mRNA derived from an intron-containing transcript	1.40E-02	
	Melanoma	8.40E-03	HGF, PIK3R2, TP53
	ECM proteoglycans	1.20E-02	DSPP, MUSK, TNC
T2B	Melanoma	3.10E-03	HGF, PIK3R2, TP53
T3B	Ribosome biogeneisis in eukaryotes	4.00E-03	BMS1, HEATR1, NXF1, REXO1
	ECM-receptor interaction	2.80E-03	COL11A1, GP1BA, THBS2, TNC
	Melanoma	7.50E-03	HGF, PIK3R2, TP53
T4B	NEP/NS2 interacts with the cellular export machinery	4.10E-04	NUP153, NUP210, XPO1
	Rev-mediated nuclear export of HIV RNA	3.00E-04	
	Export of viral ribonucleoproteins from nucleus	3.40E-04	
	Interactions of Rev with host cellular proteins	2.00E-04	
M	Prolactin signaling pathway	6.30E-03	CSN2, ESR1, PIK3R2
	Melanoma	1.20E-02	HGF, PIK3R2, TP53
	Muscle contraction	6.70E-03	MYL9, NEB, TPM2

Abbreviations: ESCC, esophageal squamous cell carcinoma; PtA, patient A; PtB, patient B.

## Supplementary Material

Supplementary Informations

Supplementary Figure S2

Supplementary Figure S3A

Supplementary Figure S3B
