# Supplementary material for: Multiple region whole-exome sequencing reveals dramatically evolving intratumor genomic heterogeneity in esophageal squamous cell carcinoma
Source: Oncogenesis. 2015 Nov 30;4(11):e175–. doi: 10.1038/oncsis.2015.34 (PMC4670960; doi:10.1038/oncsis.2015.34)
Supplement: Supplementary Informations [file oncsis201534x1.docx]

Supplementary Information for

**Multiregion whole-exome sequencing reveals dramatically evolving intratumor genomic heterogeneity in esophageal squamous cell carcinoma**

Table S1. Patients basic information

Table S2. List of non-silent mutations in all tumor regions of PtA

Table S3. List of non-silent mutations in all tumor regions of PtB

Table S4. Gene list for independent validation of mutations by Sanger sequencing

Figure S1. The classification of all mutations from multiple regional whole exome sequencing in ESCC

Figure S2. Mutational spectra from multiple region exome sequencing in ESCC

Figure S3A & S3B. The heatmap of copy number alterations in each ESCC tumor region from PtA and PtB

Figure S4. The scheme of functional analysis for non-silent mutations derived from multiple region whole exome sequencing
