# Supplementary material for: Multiple region whole-exome sequencing reveals dramatically evolving intratumor genomic heterogeneity in esophageal squamous cell carcinoma
Source: Oncogenesis. 2015 Nov 30;4(11):e175–. doi: 10.1038/oncsis.2015.34 (PMC4670960; doi:10.1038/oncsis.2015.34)
Supplement: Supplementary Table S1 [file oncsis201534x2.doc]

| Code | ID | Gender | Age | Histopathological diagnosis | Clinical stage | Treatment |
| --- | --- | --- | --- | --- | --- | --- |
| PtA | 1228350 | Male | 61 | ESCC at upper esophagus, lymph nodes invasion (0/8) | T2N0M0 | Surgery and chemotherapy |
| PtB | 201209948 | Male | 69 | Invasive ESCC at upper esophagus, lymph nodes invasion (2/6) | T3N2M0 | Surgery and chemotherapy |

**Supplementary Table S1.** Basic information of subjects with esophageal squamous cell carcinoma (ESCC)
