# Supplementary figures and images for: Multiple region whole-exome sequencing reveals dramatically evolving intratumor genomic heterogeneity in esophageal squamous cell carcinoma
Source: Oncogenesis. 2015 Nov 30;4(11):e175–. doi: 10.1038/oncsis.2015.34 (PMC4670960; doi:10.1038/oncsis.2015.34)

**Figure S1**

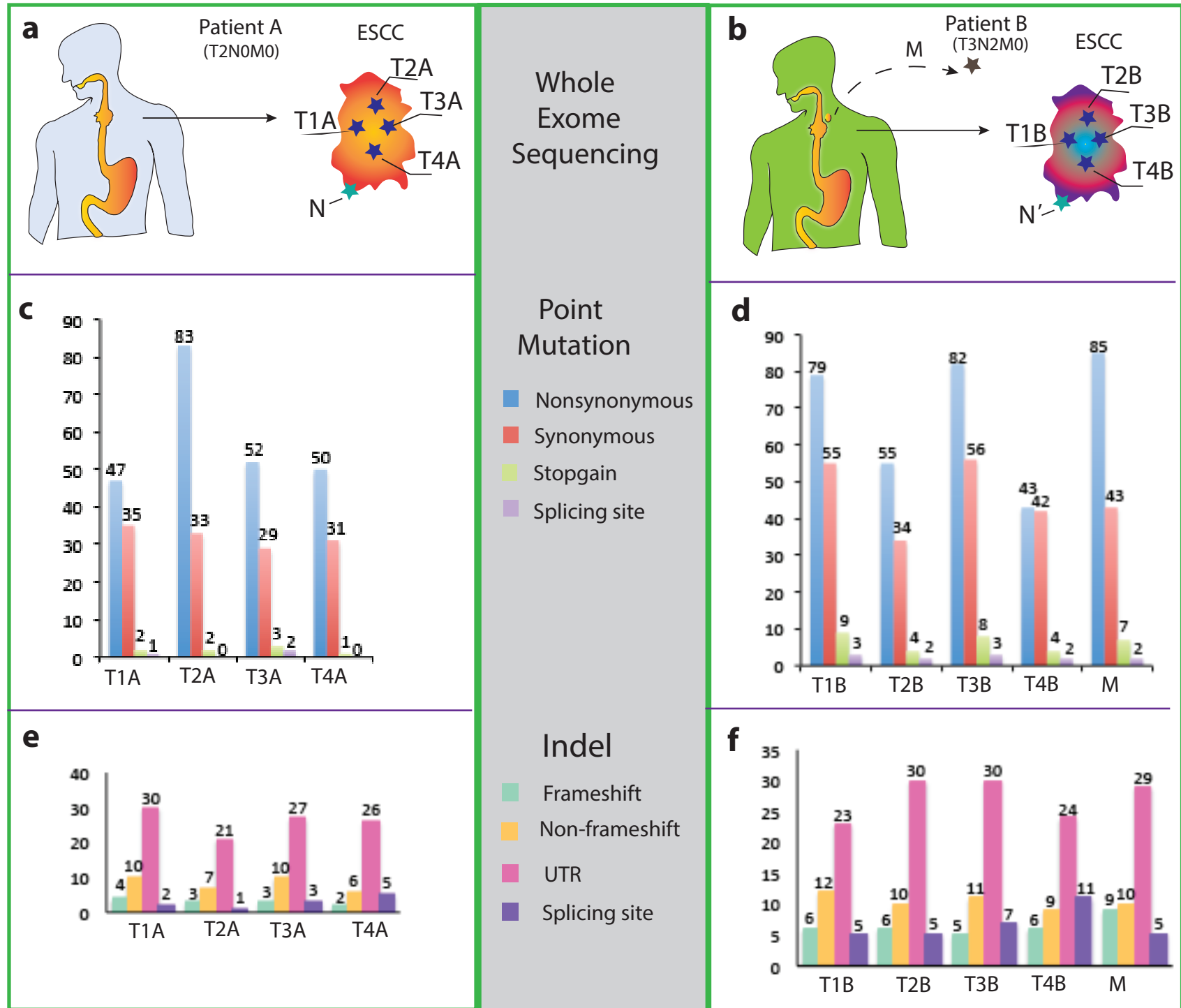

Supplement: Supplementary Figure S1 [file oncsis201534x6.pdf]

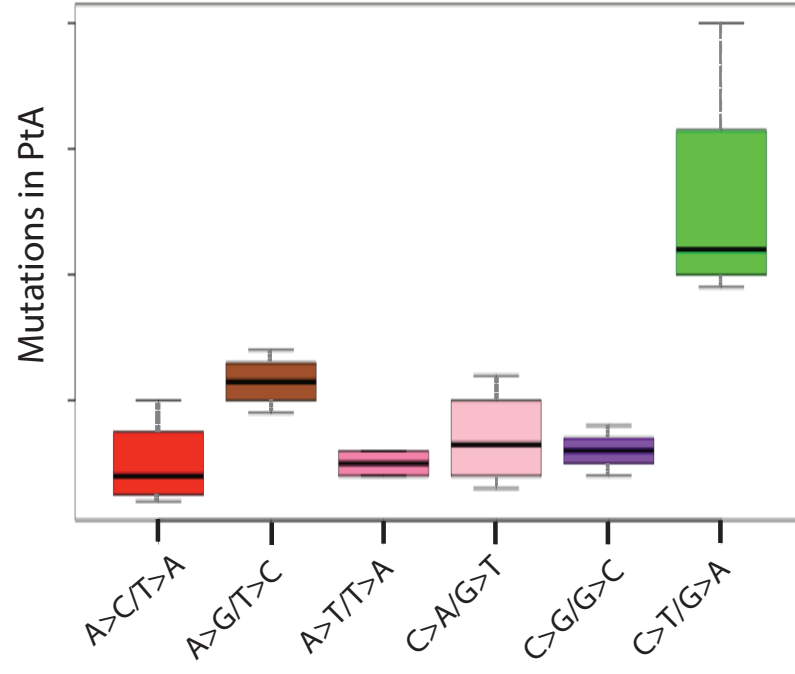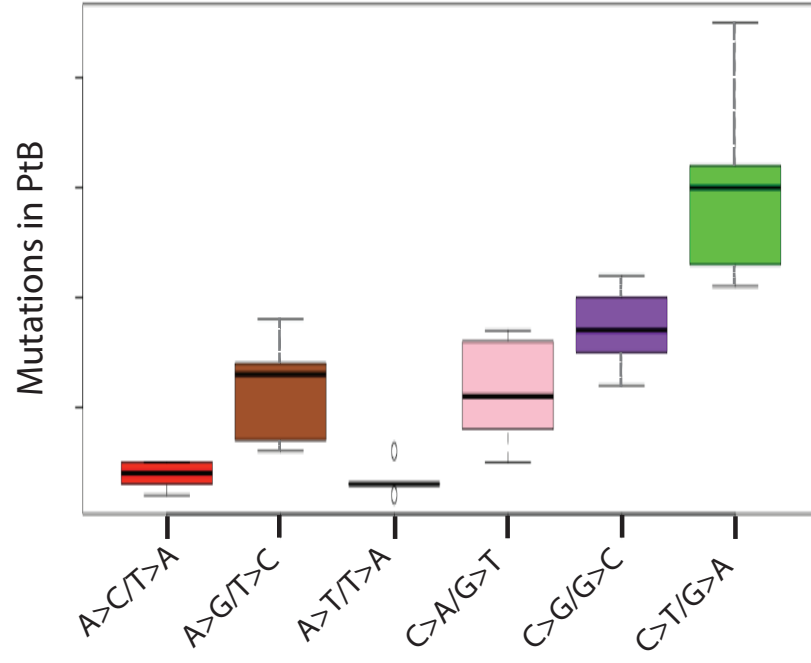

Supplement: Supplementary Figure S2 [file oncsis201534x7.pdf]

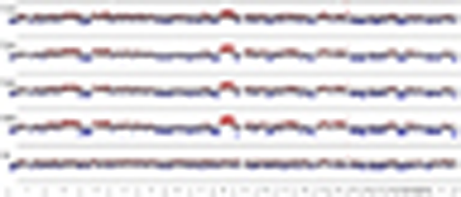

Supplement: Supplementary Figure S3A [file oncsis201534x8.tif]

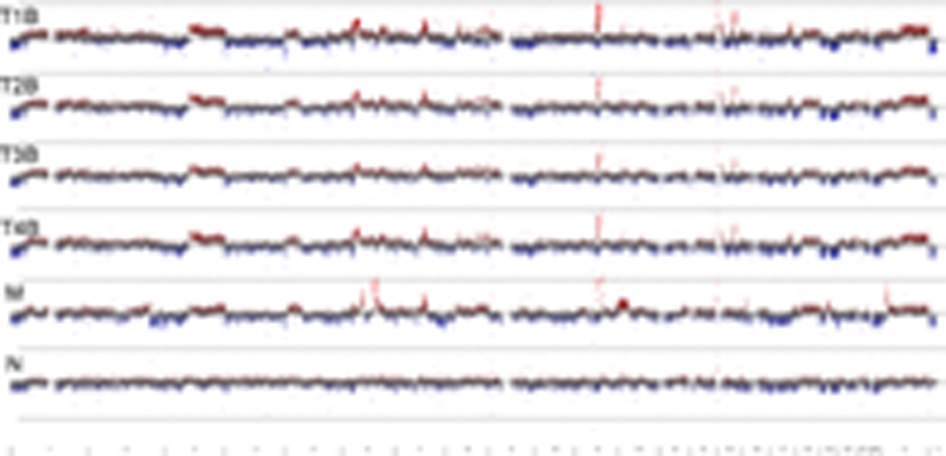

Supplement: Supplementary Figure S3B [file oncsis201534x9.tif]

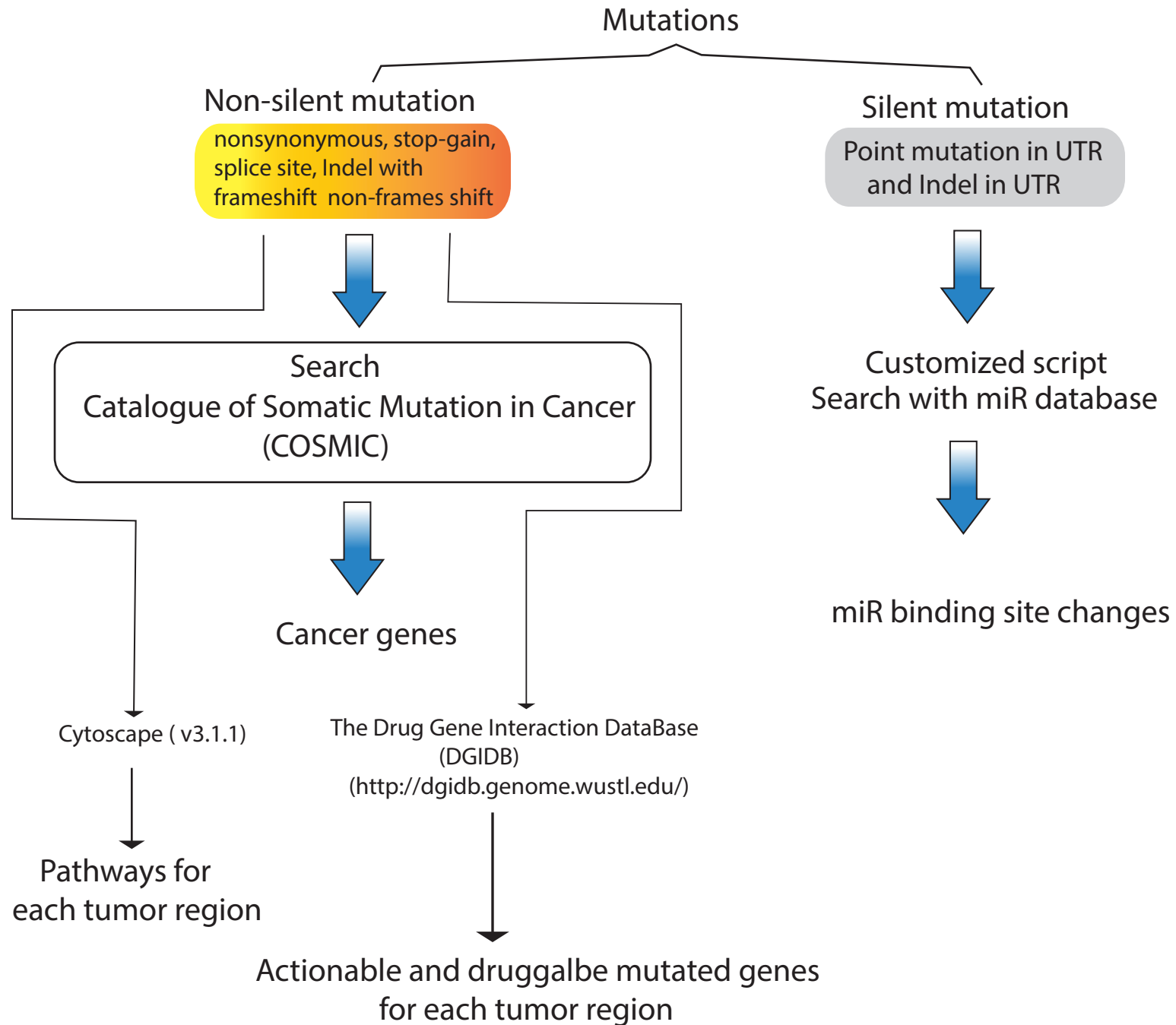

Supplement: Supplementary Figure S4 [file oncsis201534x10.pdf]
